# Multipole Radiations from Large Gold Nanospheres Excited by Evanescent Wave

**DOI:** 10.3390/nano9020175

**Published:** 2019-01-31

**Authors:** Jingdong Chen, Jin Xiang, Shuai Jiang, Qiaofeng Dai, Shaolong Tie, Sheng Lan

**Affiliations:** 1 Guangdong Provincial Key Laboratory of Nanophotonic Functional Materials and Devices, School of Information and Optoelectronic Science and Engineering, South China Normal University, Guangzhou 510006, China; jdchen@163.com (J.C.); xiangcos@163.com (J.X.); jiangs1224@163.com (S.J.); daiqf@scnu.edu.cn (Q.D.); 2 College of Physics and Information Engineering, Minnan Normal University, Zhangzhou 363000, China; 3 School of Chemistry and Environment, South China Normal University, Guangzhou 510006, China; tiesl@scnu.edu.cn

**Keywords:** gold nanosphere, electric dipole, electric quadrupole, electric octupole, plasmon mode, scattering, evanescent wave, radiation pattern

## Abstract

We proposed the use of the evanescent wave generated in a total internal reflection configuration to excite large gold nanospheres and investigated the radiations of the high-order plasmon modes supported in gold nanospheres. It was revealed that the evanescent wave excitation is equivalent to the excitation by using both the incident and reflected light, offering us the opportunity to control the orientation of the electric field used to excite nanoparticles. In addition, it was found that the scattering light intensity is greatly enhanced and the background noise is considerably suppressed, making it possible to detect the radiations from high-order plasmon modes. Moreover, the influence of the mirror images on the scattering induced by a metal substrate is eliminated as compared with the surface plasmon polariton excitation. By exciting a gold nanosphere with *s*-polarized light and detecting the scattering light with a *p*-polarized analyzer, we were able to reveal the radiation from the electric quadrupole mode of the gold nanosphere in both the spatial and the frequency domains. Our findings are important for characterizing the radiations from the high-order modes of large nanoparticles and useful for designing nanoscale photonic devices.

## 1. Introduction

Surface plasmons supported in noble metallic nanostructures have formed a highly attractive branch of nanoscale optical physics in the last two decades. Due to the excitation of collective coherent quantized oscillations of conduction band electrons in the metal nanostructures, both the absorption and scattering cross-sections are significantly enlarged compared with their physical dimensions, resulting in a strong light–matter interaction, subwavelength volume confinement, and optical field enhancement [1,2]. Owing to the superior properties of surface plasmons, various potential applications have been inspired including surface-enhanced Raman spectroscopies [3,4,5,6,7], ultrasensitive biosensing [8,9,10], fluorescence enhancement [11,12], photothermal therapy [13,14,15], ultrahigh density data storage [16,17], nonlinear optics conversion [18,19,20,21], and quantum optics [22], etc.

Of the various nanostructures, spherical nanoparticles are one of the most fundamental and important ones that possess the highest geometrical symmetry and could act as the simplest isotropic antenna. More interestingly, one can construct more complicated and sophisticated nanostructures (e.g., dimers, oligomers, and particle-on-film [23,24,25,26,27,28,29,30,31,32,33,34]) which function as photonic devices by using spherical nanoparticles as building blocks. Based on Mie theory [35], the far-field scattering of a plasmonic spherical particle can be analytically decomposed into the contributions of multipoles. With increasing diameter, the electric dipole (ED) of the nanoparticle will be redshifted to the near-infrared spectral range while the high-order plasmon modes, such as electric quadrupole (EQ) and electric octupole (EOC), will emerge in the visible light spectrum. For instance, the high-order plasmon modes of spherical gold mesoparticles are experimentally observed by using reflectance spectroscopy [36]. Owing to the broad linewidth of these plasmon modes, the coherent interaction between them can be employed to realize highly directional scattering, delicate beam shaping, and effective wavefront control [34,37,38]. For example, it was shown that highly directional plasmonic nanoantennas can be realized by exploiting the constructive interference between the ED and EQ of a nano-ring when the generalized Kerker condition is satisfied [38,39]. This intriguing behavior indicates the crucial role of the high-order plasmon modes in engineering the far-field radiation in the nanoscale plasmonic structures. Owing to the inherent Ohmic loss of metals, the linewidth of the ED resonance is much broader than those of the high-order plasmon modes, implying the importance of the high-order plasmon modes in practical applications where an enhancement in the electric field is required, such as surface-enhanced hyper-Raman scattering [6,7]. Therefore, the investigation of the high-order plasmon modes induced in metallic nanospheres, especially the EQ mode, is quite important for designing photonic devices capable of manipulating light radiation and emission.

So far, dark-field microscopy is the most popular way to characterize the far-field scattering of nanoparticles. Although it is a simple and effective method to measure the forward/backward scattering of nanoparticles, there are still some limitations. For example, an objective with a small numerical aperture (NA) is generally employed in a dark-field microscope to collect the scattering light in order to exclude the illumination light. Thus, only a small fraction of the scattering light is detected, leading to a weak scattering intensity or a low signal-to-noise ratio. Moreover, it is not convenient to control the polarization of the illumination light and thus the orientation of the electric field used to excite the nanoparticle. In our previous work [40], we used the surface plasmon polaritons generated on the surface of a thin gold film to excite the gold nanospheres (GNSs) placed on the thin film and investigated the radiation behaviors of various plasmon modes excited in large GNSs. In this case, the scattering intensity of a GNS is greatly enhanced due to the strongly localized electric field in the surface plasmon polaritons, offering us the opportunity to investigate the radiations from the high-order plasmon modes, such as the electric quadrupole (EQ) and electric octupole (EOC) modes. In addition, a completely dark background is achieved in the total internal reflection configuration utilized to excite the surface plasmon polaritons, resulting in radiation patterns with high contrasts. Unfortunately, there are still three drawbacks in this method. Since the surface plasmon polaritons can only be created by using p-polarized, the polarization control of the illumination light is strongly limited. Moreover, the existence of the thin gold film will introduce mirror images for all plasmon modes and the scattering of a GNS is finally determined by the coherent interaction of the plasmon modes and their mirror images, making the spatial and frequency analysis of the scattering behavior more complicated. Finally, two gap plasmon modes, which are oriented horizontally and vertically, are introduced in between the GNS and the thin gold film and contribute to the total scattering of the GNS. Apart from conventional dark-field microscopy, scanning nearfield optical microscopy [41,42], photoelectron emission microscopy [43,44,45], and electron microscopy [46,47,48] have also been employed for optical field imaging at the nanoscale and even spectrum recording.

Very recently, we proposed the use of the evanescent wave generated in the total internal reflection configuration to excite silicon nanoparticles for a color-tuning display with a high spatial resolution and a good chromaticity [49]. In this strategy, the orientations of the electric and magnetic fields used to excite the Si nanoparticle can be conveniently adjusted by simply varying the polarization angle of the incident light. The excitation of a nanoparticle by using such an evanescent wave is equivalent to the excitation of the nanoparticle by using both the incident and the reflected light. The scattering intensity of the nanoparticle is significantly enhanced while the background noise is considerably suppressed. Compared to the surface plasmon polaritons, the influence of the mirror images is eliminated and the polarization control of the excitation light becomes possible and convenient.

In this article, we employed the evanescent wave generated in the total internal reflection configuration to excite the GNSs placed on a silica substrate. The high-order plasmon modes excited in the GNS were investigated by exciting the GNSs with s- and p-polarized light and detecting the scattering light of the GNSs with a cross-polarized analyzer. We revealed the radiation from the quadrupole of a large GNS in both frequency and spatial domains by using *s*-polarized evanescent wave excitation in combination with a cross-polarized analyzer in the collection channel. We also indicate that highly directional radiation may be realized by exploiting the coherent interaction between the ED and EQ modes of the GNS.

## 2. Experimental and Numerical Methods 

### 2.1. Sample Preparation

**Figure 1:**
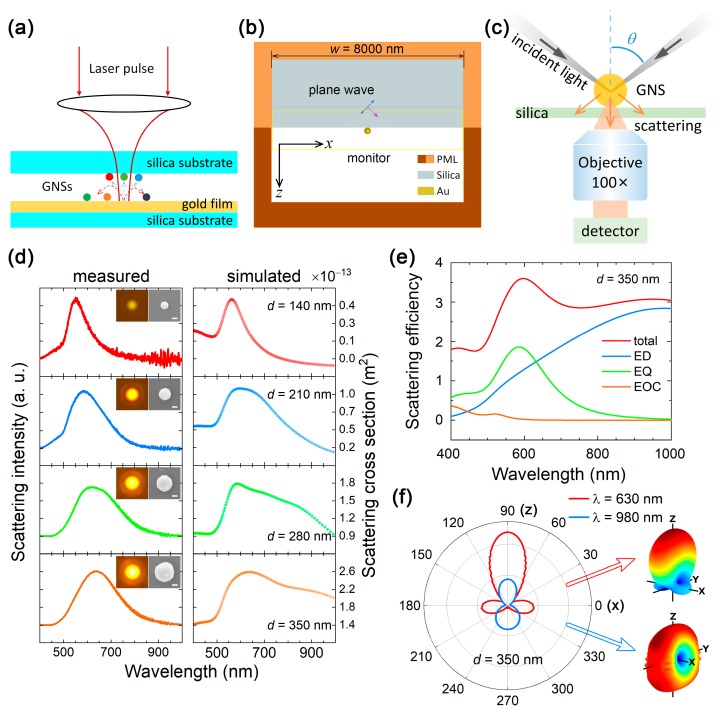
(**a**) Schematic showing the fabrication of gold nanospheres (GNSs) with different diameters on a silica substrate by using fs laser ablation. (**b**) Configuration used to simulate the far-field radiation from a GNS excited by an evanescent wave in the finite-difference time-domain (FDTD) simulations. (**c**) Schematic showing the illumination of a GNS placed on a silica substrate with white light at an incidence angle of *θ* ~58°, and the detection of the forward scattering light in a dark-field microscope. (**d**) Forward scattering spectra measured and simulated for GNSs with different diameters placed on a silica substrate. The SEM images of the corresponding GNSs and the coupled charge device (CCD) images of the scattering light are shown in the insets. The length of the scale bar is 100 nm in all cases. (**e**) The total scattering spectrum calculated for a GNS with *d* = 350 nm in free space based on Mie theory. The scattering has been decomposed into the contributions of electric dipole (ED), electric quadrupole (EQ), and electric octupole (EOC). (**f**) Two- and three-dimensional radiation patterns calculated for the GNS with *d* = 350 nm in free space at two wavelengths of 630 and 980 nm.

We employed femtosecond (fs) laser ablation to fabricate GNSs with diameters ranging from 80–560 nm. In experiments, as schematically shown in Figure 1a, 800-nm fs laser light delivered by a fs amplifier (Legend Elite, Coherent, Santa Clara, CA, USA) with a pulse duration of 90 fs and a repetition rate of 1 kHz was focused on the surface of a 50-nm-thick Au film by using a lens with 22-mm focal length. The ejected GNSs with different diameters were randomly distributed on a silica substrate covered on the Au film. The morphologies and sizes of the GNSs were examined by using a scanning electron microscope (SEM) (Ultra55, Zeiss, Stutt-gart, Germany).

### 2.2. Optical Characterization

The scattering properties of GNSs excited either by using the white light in a conventional dark-field microscope or by using the evanescent wave generated in a total internal reflection configuration were characterized by using an inverted microscope (Axio Observer A1, Zeiss, Stutt-gart, Germany) equipped with a spectrometer (SR-500i-B1, Andor, Oxford, England) and a full-color coupled charge device (CCD) (DS-Ri2, Nikon, Tokyo, Japan). In the conventional dark-field microscopy, the white light was incident on the Au film at an angle of ~58° with respect to the normal of the surface. In comparison, *s*- and *p*-polarized white light was launched into a prism at an incidence angle of ~45° to excite the evanescent wave. The silica substrate was attached on the bottom surface of the prism with silicon oil for refractive index matching. The scattering light of GNSs was collected with a 100× objective (NA = 0.7−1.3, Plan-NEOFWAR, Zeiss, Stutt-gart, Germany) in the forward direction and analyzed by inserting a polarization analyzer in the collecting channel.

### 2.3. Numerical Modeling

The scattering spectra of GNSs were either analytically calculated based on Mie theory or numerically simulated by using the finite-difference time-domain (FDTD) technique. The multipolar contributions to the total scattering of the GNSs are calculated analytically and numerically. The permittivity of Au was fitted from experimental data [50]. For the simulation of the scattering spectra of GNSs excited by the white light in the conventional dark-field microscopy, we used a total-field/scattered-field source to simulate the scattering efficiencies of GNSs excited by a plane wave incident normally on the silica substrate. Differently, a plane wave with an incidence angle of ~45° was employed to simulate the scattering spectra of GNSs excited by the evanescent wave, as schematically shown in Figure 1b. In the numerical simulations, evanescent waves were automatically created on the surface of the silica substrate when the condition of total internal reflection was satisfied. A cubic monitor with a volume of 8000 (length) × 8000 (width) × 1600 (height) nm^3^ enclosing the GNSs was used to record the electromagnetic field distributions. A standard two-step method in which the total scattering field and background field in the absence of GNSs were extracted separately was employed to calculate the net scattering fields of GNSs excited by the evanescent waves. Based on the net scattering fields, one can obtain the far-field scattering properties of GNSs under the excitation of the evanescent wave. In order to compare the simulation results with the experimental observations, we detected only the scattering in the forward direction by using a monitor with an area of ~1000 (length) × 1000 (width) nm^2^ in the calculation of the scattering cross-section which offers a collection angle quite similar to that provided by the objective with NA = 0.7 and index-match oil (~1.4). Differently, the net electromagnetic field distributions from all six monitors were extracted to obtain the far-field radiation patterns. Based on the calculated scattering fields and the Poynting vectors, in addition to their components, the scattering cross-sections and far-field radiation patterns of GNSs excited by the evanescent with or without a polarization analyzer could be obtained. The mesh size used in the GNS was set to be smaller than 1/60 of its radius. A perfectly matched layer boundary condition was employed to terminate the finite simulation region.

## 3. Results and Discussion

### 3.1. Dark-Field Microscopy

The scattering properties of large GNSs prepared by using fs laser ablation were firstly examined by conventional dark-field microscopy, as schematically shown in Figure 1c. The scattering spectra measured for GNSs with diameters ranging from 140 to 350 nm are presented in Figure 1d. The simulation results based on the FDTD technique in which the silica substrate is taken into account are also provided for comparison. The SEM images of the GNSs and the radiation patterns of the GNSs recorded by using a CCD are shown as insets.

Based on Mie theory, the EQ and EOC modes emerge in the visible light spectrum when the diameter of the GNS becomes larger than 220 and 380 nm, respectively. Since the refractive index of silica is small (~1.45), its influence on the multipole resonances of Au nanospheres is quite small. In Figure 1e, we show the scattering spectrum analytically calculated for the GNS with *d* = 350 nm based on Mie theory. It has been decomposed into the contributions of ED, EQ, and EOC modes. It is noticed that the ED and EQ modes of the GNS appear at ~980 and 585 nm, respectively. Owing to the large linewidths of the ED and EQ resonances, the spectral overlapping of the ED and EQ modes occurs in a wide wavelength range of 500–820 nm. It implies that the scattering of the GNS in this wavelength range is governed by the coherent interaction of the ED and EQ modes. As a consequence, the forward scattering at the EQ resonance becomes stronger than that at the ED resonance, see Figure 1d. In Figure 1f, we present the 2D and 3D radiation patterns of the GNS calculated at two wavelengths of 980 and 630 nm. At 980 nm where the ED resonance is located, we observed a radiation pattern of a typical ED mode oriented along the *x* direction; although, it is slightly distorted due to the existence of a small amount of the EQ mode. In this case, the intensities of the forward and backward scattering are almost the same. In sharp contrast, it is found that the forward scattering is significantly enhanced while the backward scattering is greatly suppressed at 630 nm where the amplitudes of the ED and EQ resonances are equal. The highly directional radiation achieved in the forward direction originates from the constructive interference between the ED and EQ modes. The simulated radiation pattern at ~630 nm appears to be quite similar to that obtained by the constructive interference between a dipole and a quadrupole, verifying the physical mechanism for the enhanced forward scattering.

In Figure 1d, the scattering spectra measured for GNSs are in good agreement with those predicted based on numerical simulations except for the wavelengths larger than 800 nm where the quantum efficiency of the detector drops rapidly. By increasing the diameter of the GNS, one can see a redshift of the scattering peak and a broadening of the scattering spectrum. Since the redshift of the ED resonance is larger than that of the EQ resonance, the coherent interaction between them will become weaker in large GNSs. For the smallest GNS with *d* = 140 nm, the scattering spectrum is governed by the ED resonance and the scattering light appears as green-yellow. For the GNSs with *d* > 220 nm, the ED resonance is redshifted to long wavelengths and the scattering spectrum is dominated by the EQ resonance appearing in the wavelength range of 550–650 nm. Since the contribution of the ED resonance to the scattering light is still large, the color of the scattering light appears as white. In all cases, the radiation pattern of the GNS appears to be the typical one of a horizontally oriented ED mode (i.e., a bright spot) although it contains the contribution from the EQ mode. It means that the scattering light from the EQ mode cannot be resolved by using conventional dark-field microscopy.

### 3.2. Evanescent Wave Excitation

**Figure 2:**
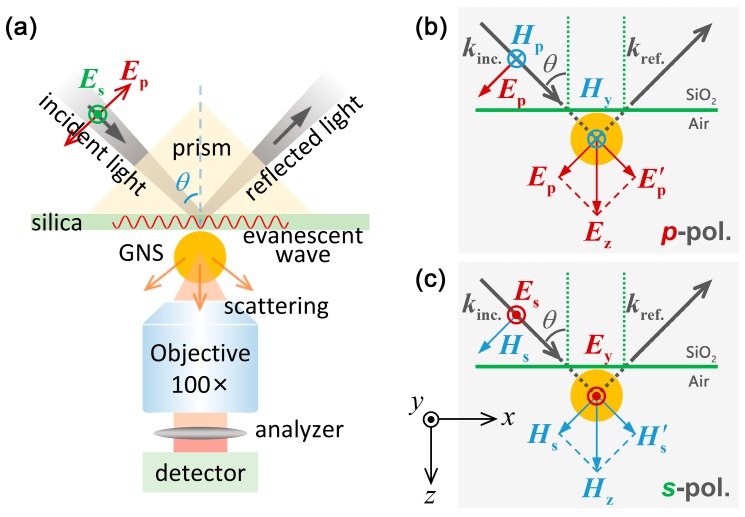
(**a**) Schematic showing the excitation of a GNS with the evanescent wave generated in a total internal reflection configuration and the detection of the quadrupole radiation of the GNS by filtering out the dipole radiation with a cross-polarized analyzer. The incidence angle is chosen to be *θ* ~ 45°. (**b**) Schematic showing the excitation of a GNS by using the evanescent wave generated with *p*-polarized light. (**c**) Schematic showing the excitation of a GNS by using the evanescent wave generated with *s*-polarized light. In (**b**) and (**c**), the combined electric and magnetic fields of the incident and reflected light which determine the orientations of the ED and magnetic dipole (MD) induced in the GNS by *p*- and *s*-polarized light are illustrated.

In order to gain a deep insight into the radiation of the EQ mode of a large GNSs, we employed the evanescent wave generated in a total internal reflection configuration to excite the GNS and detected the forward scattering of the GNS with the help of a polarization analyzer, as schematically shown in Figure 2a. The advantages of evanescent wave excitation over the conventional dark-field microscopy are threefold. First, one can choose to excite the GNS with either *p*- or *s*-polarized light, making it possible to manipulate the orientation of the electric field used to excite the GNS. Second, the evanescent wave excitation is basically equivalent to the excitation of GNS with both the incident and reflected light with a fixed phase difference. The in-phase overlap of the electric fields of the two-excitation light at the critical angle for total internal reflection leads to a vertically oriented electric field for *p*-polarized light and a horizontally oriented electric field for *s*-polarized light, as shown in Figure 2b,c, respectively. In both cases, the scattering light is enhanced while the background noise is suppressed, improving significantly the signal-to-noise ratio in the measurements. Finally, the excitation of the GNS with polarized light offers the possibility to completely eliminate the radiation of the ED mode by inserting a cross-polarized analyzer into the collection channel [49,51,52], making it possible to resolve the quadrupole scattering in both the frequency and spatial domains.

In the experiments, the incidence angle was chosen to be the critical angle for total internal reflection (~45°) so that the reflected light is in phase with the incident one (i.e., the phase difference is approximately zero). In this case, the electric field of the evanescent wave induced by the *s*-polarized light has only the horizontal component (*E*_y_) while that induced by the *p*-polarized light has only the vertical component (*E*_z_). In this way, we can study the scattering properties of a large GNS excited by a horizontally or vertically aligned electric field.

**Figure 3:**
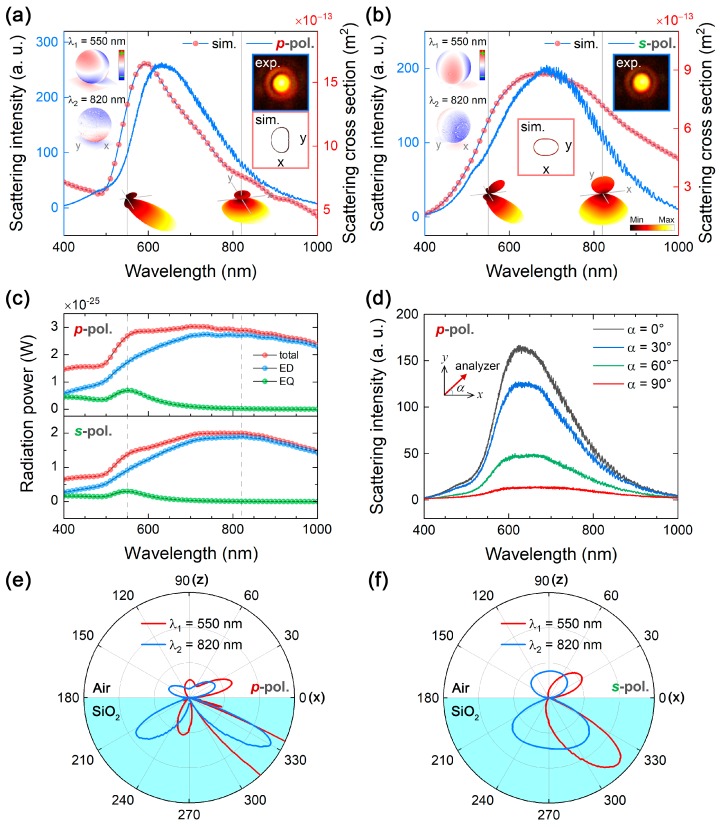
Scattering spectra measured and simulated for a GNS with *d* ~ 280 nm excited by (**a**) *p*- and (**b**) *s*-polarized light. Insets show the calculated three-dimensional far-field radiation patterns at 550 and 820 nm. The surface charge distributions at the two wavelengths are also provided as insets. (**c**) Multipole expansion of the total scattering of a GNS with *d* = 280 nm excited by using *p*- and *s*-polarized light. (**d**) Scattering spectra of the GNS with *d* ~ 280 nm excited by *p*-polarized light and measured by setting the polarization analyzer at different angles (α) of 0°, 30°, 60°, and 90°. The two-dimensional radiation patterns (on the *xoz* plane) calculated for the GNS excited by *p-* and *s*-polarized light are shown in (**e**) and (**f**), respectively.

In Figure 3, we show the scattering spectra measured and simulated for a GNS with *d* ~ 280 nm which is excited by using *p*- and *s*-polarized light. When the GNS is excited by using *p*-polarized light, the peak of the scattering spectrum appears at ~620 nm. In comparison, the scattering peak of the GNS is redshifted to ~690 nm with a small shoulder at ~570 nm when *s*-polarized is used. It is also noticed that the simulated spectrum under the excitation of *s*-polarized light, shown in Figure 3b, appears to be similar to the one obtained in the conventional dark-field microscopy, see Figure 1d. This is because the excitation of the GNS achieved by using the evanescent induced by s-polarized light is actually the excitation of the GNS with a horizontally aligned electric field, which is exactly the same as the excitation of the GNS by using a normally incident plane wave. If we compare the scattering patterns recorded by using a CCD in the two cases, however, it is found that the contrast of the CCD image obtained in the evanescent wave excitation is much better than that obtained in the dark-field microscopy. The significantly improved contrast of the CCD image arises from the considerably suppressed background in the generation of the evanescent wave where the total internal reflection configuration is employed.

### 3.3. Multipole Expansion of the Scattering Spectrum

Since the refractive index of silica is close to that of a vacuum, the existence of the silica substrate has a negligible influence on the plasmon modes excited in a GNS. Since the phase difference between the incident and reflected light is zero at the critical angle of the total internal reflection, the excitation of the GNS by using an evanescent wave is equivalent to the simultaneous excitation of the nanoparticle with two plane waves. Therefore, we employed the multipole expansion method to approximately determine the electric resonances in a GNS excited by an evanescent wave in order to understand the physical origins of the scattering peaks appearing in the scattering spectra of the GNS shown in Figure 3a,b. In a Cartesian coordinate, the radiated moments of electric dipole (**ED**), magnetic dipole (**MD**), toroidal dipole (**TD**), electric quadrupole (**EQ**), and magnetic quadrupole (**MQ**) can be derived from the induced volume current density **j** in the GNS as follows in the SI units [53,54,55,56]:

(1) $$\mathrm{ED}=\frac{1}{i\omega }\int jd^{3}r$$

(2) $$\mathrm{MD}=\frac{1}{2c}\int \left(r\times j\right)d^{3}r$$

(3) $$\mathrm{TD}=\frac{1}{10c}\int \left[\left(r\cdot j\right)r-2r^{2}j\right]d^{3}r$$

(4) $$EQ_{\alpha \beta }=\frac{1}{i\omega }\int \left[r_{\alpha }j_{\beta }+r_{\beta }j_{\alpha }-\frac{2}{3}\left(r\cdot j\right)\right]d^{3}r$$

(5) $$MQ_{\alpha \beta }=\frac{1}{3c}\int \left[{\left(r\times j\right)}_{\alpha }r_{\beta }+{\left(r\times j\right)}_{\beta }r_{\alpha }\right]d^{3}r$$

Here, *c* is the speed of light in vacuum and *α*, *β* = *x*, *y*, *z*. The total radiating power can then be expressed as:

(6) $$\begin{array}{l} I=\frac{2\omega^{4}}{3c^{3}}{\left|\mathrm{ED}\right|}^{2}+\frac{2\omega^{4}}{3c^{3}}{\left|\mathrm{MD}\right|}^{2}+\frac{4\omega^{5}}{3c^{4}}\left(\mathrm{ED}\cdot \mathrm{TD}\right)\\ +\frac{2\omega^{6}}{3c^{5}}{\left|T\right|}^{2}+\frac{\omega^{6}}{5c^{5}}\sum {\left|EQ_{\alpha \beta }\right|}^{2}+\frac{\omega^{6}}{40c^{5}}\sum {\left|MQ_{\alpha \beta }\right|}^{2} \end{array}$$

We show the decomposed scattering spectra of a GNS with *d* = 280 nm excited by using p-and s-polarized light in Figure 3c. For clarity, only the ED and EQ resonances are presented because the contributions of other plasmon modes are negligible. It can be seen that the total scattering of the GNS is dominated by the ED mode with a broad linewidth and a peak at ~820 nm. In comparison, the EQ mode possesses a narrower linewidth and a weak intensity. It is located at ~550 nm, which corresponds to the small shoulder in the scattering spectrum. As compared with the simulated spectra shown in Figure 3a,b, it is noticed that the ED resonance is stronger in the near-infrared spectral range because the scattering intensities in all directions are taken into account in the multipole expansion method. In contrast, only the forward scattering is considered in the simulated spectra shown in Figure 3a,b. In this case, highly directional scattering is observed at the EQ resonance when the generalized Kerker condition [39] is satisfied.

### 3.4. Revealing High-Order Plasmon Modes

Based on the multipole decomposition shown in Figure 3c, we were able to determine the peak wavelengths of the EQ and ED modes in the GNS with *d* = 280 nm, which are located at ~550 and ~820 nm (labeled as $\lambda_{1}$ and $\lambda_{2}$, respectively). Surface charge distributions calculated for the GNS at the ED and EQ resonances are presented in the insets of Figure 3a,b for *p*- and *s*-polarized light excitations. For the *p*-polarized light excitation, a vertically aligned ED mode is identified from the surface charge distribution on the GNS at 820 nm. In addition, a typical EQ mode is revealed from the surface charge distribution at 550 nm. For the *s*-polarized light excitation, the surface charge distribution at 820 nm indicates a horizontally aligned ED mode, while that at 550 nm displays a horizontally oriented EQ mode. The surface charge distributions on the GNS at the ED and EQ resonances agree well with the combined electric fields induced by the incident and reflected light shown in Figure 2b,c. It verifies that a horizontally and vertically aligned electric field can be selectively created by using the evanescent wave generated in the total internal reflection configuration. The orientations of the plasmon modes can be experimentally identified by analyzing the polarization of the scattering light. For example, the scattering intensities of the ED and EQ modes of the GNS with *d* ~ 280 nm excited by *p*-polarized light decreased as the angle of the polarization analyzer was increased from 0° to 90°, as shown in Figure 3d. However, the ED mode located at a longer wavelength was not completely removed because it orients vertically.

In Figure 3e,f, we present the two-dimensional (2D) far-field radiation patterns on the *xoz* plane calculated at wavelengths of 820 and 550 nm for *p*- and *s*-polarized light. The three-dimensional (3D) radiation patterns are also provided in the insets of Figure 3a,b. In Figure 3a, the radiation pattern at $\lambda_{2}$ = 820 nm is dominated by a vertically oriented ED. Owing to the existence of the silica substrate, the radiation intensity into the substrate is much stronger than that into air. At $\lambda_{1}$ = 550 nm, the radiation pattern is determined by the coherent interaction between EQ and ED modes. From the 2D pattern on the *xoz* plane shown in Figure 3e, it can be seen that the scattering light in the +*z* direction (from 60° to 120°, corresponding to the NA of the objective) is dominated by the EQ mode. As a result, the calculated and simulated scattering spectra are dominated by the EQ mode rather than the ED mode when the GNS is excited by using *p*-polarized light, as shown in Figure 3a. In contrast, the radiation pattern of the ED mode is dramatically changed when the GNS is excited by using *s*-polarized light, as shown in the inset of Figure 3b. The existence of the silica substrate significantly modifies the radiation of a horizontally oriented ED mode, see the inset of Figure 1f. The radiation pattern is quite similar to the result reported previously [34]. In addition, the radiation of the EQ mode in the +*z* direction is greatly reduced, as clearly shown in Figure 3f. In this case, only a small fraction of the EQ mode is collected in both the measurement and the simulation. Consequently, the measured scattering spectrum is dominated by the ED mode rather than the EQ mode because the collection angle of the objective is ~60°, as reflected in Figure 3b. 

**Figure 4:**
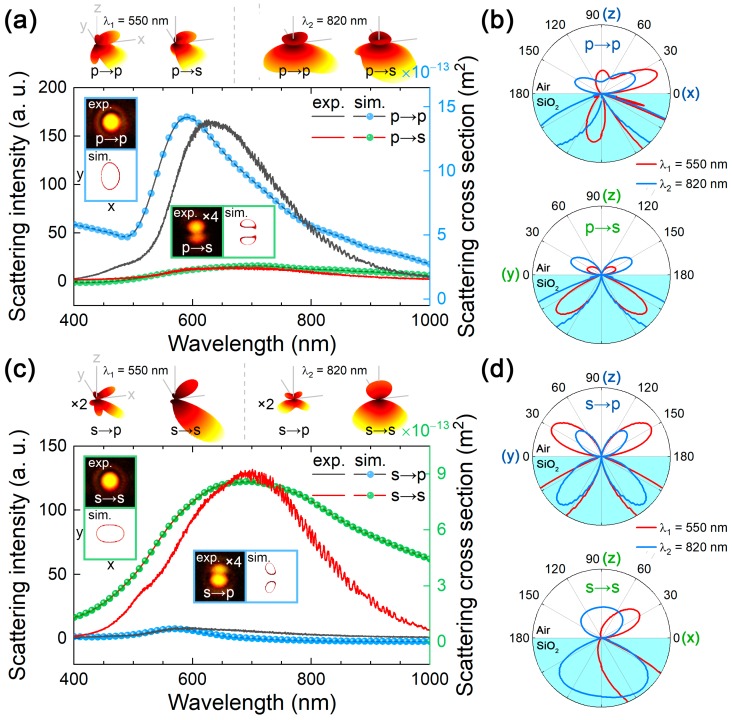
Scattering spectra measured and simulated for a GNS with *d* ~ 280 nm, which is excited by using *p*- and *s*-polarized light and detected by using a *p*- and *s*-polarized analyzer. (**a**) Excited by *p*-polarized light and detected by using a *p*- and *s*-polarized analyzer. (**b**) Two-dimensional radiation patterns on the *xoz* and *yoz* planes of the GNS excited by using *p*-polarized light at 550 and 820 nm and detected by using a *p*- and *s*-polarized analyzer. (**c**) Excited by *s*-polarized light and detected by using a *p*- and *s*-polarized analyzer. (**d**) Two-dimensional radiation patterns on the *xoz* and *yoz* planes of the GNS excited by using *s*-polarized light 550 and 820 nm detected by using a *p*-/*s*-polarized analyzer. The two-dimensional radiation patterns recorded by using a CCD and those on the *xoy* plane and the three-dimensional radiation calculated by using the FDTD method are shown in the insets of (**a**) and (**c**).

Since the scattering light comprises the radiations from the ED and EQ modes, it is necessary to eliminate the radiation of the ED mode in order to reveal the radiation properties of the EQ mode in both the spatial and frequency domains. In Figure 4a,b, we show the scattering spectra and radiation patterns measured for a GNS with *d* ~ 280 nm. In this case, the GNS was excited by *p*-polarized light and the scattering light was detected by inserting a *p*- or *s*-polarized analyzer in the collection channel. When the GNS is excited by using *p*-polarized light, only the vertical component of the electric field is left, see Figure 2b. In this case, the radiation of the excited ED exhibits a doughnut-shaped ring while that of the EQ mode exhibits a butterfly-like shape. For the vertically oriented ED ($\lambda_{2}$ = 820 nm), most of the scattering light is removed when a *p*- or *s*-polarized analyzer is used. As a result, one can see two spots along the polarization direction of the analyzer. The 2D pattern on the *xoz* or *yoz* plane is shown by the blue curve in Figure 4b. As compared with the case without the use of a polarization analyzer, see Figure 3a, the scattering intensity is reduced to some extent. For the EQ mode, the use of an *s*-polarized analyzer is completely different from the use of a *p*-polarized one. When a *p*-polarized analyzer was used, the radiation intensity was reduced but the radiation pattern remained unchanged, as shown by the red curve in the top panel of Figure 4b. The radiation pattern recorded by the CCD appeared as a bright spot, in good agreement with the simulated one. When an *s*-polarized analyzer was used, the radiation in the +*z* direction was completely removed at 550 and 820 nm, as shown in the lower panel of Figure 4b. From the 3D radiation pattern of the EQ mode shown in Figure 4a, it can be seen that the radiation in the upper part is almost eliminated. From the bottom panel of Figure 4b, it is observed that only two lobes are left in the 2D radiation pattern of the EQ mode after the use of the *s*-polarized analyzer, similar to the ED mode. In this case, the scattering intensity is significantly reduced because most of the scattering light is not collected by the objective. The radiation pattern exhibited two lobes along the *y*-axis.

The most interesting phenomenon is observed when the GNS was excited by using *s*-polarized light and the scattering light was measured by using a *p*-polarized analyzer, as shown in Figure 4c. In this case, the radiation of the excited ED mode, which is horizontally oriented, is completely filtered out by the cross-polarized analyzer inserted into the collection channel. As a result, we observed the scattering spectrum and radiation pattern of a pure EQ mode. The scattering spectrum was dominated by the EQ resonance peak at ~550 nm. Accordingly, the 2D patterns (on the *yoz* plane) at both 550 and 820 nm exhibit the radiation of a typical EQ mode because of its broad linewidth, implying that the ED mode has been completely removed. When an *s*-polarized analyzer was used, the scattering spectrum was governed by the strong radiation of the ED mode peaking at ~820 nm, as shown in the bottom panel of Figure 4d. Consequently, a bright spot was observed in the radiation which is typical for a horizontally oriented ED mode. Therefore, the observation of the radiation of a pure EQ mode can only be realized by exciting the GNS with *s*-polarized light and detecting the scattering light with a *p*-polarized analyzer.

## 4. Conclusions

In summary, we have fabricated GNSs with diameters ranging from 80 to 560 nm by using femtosecond laser ablation and proposed the use of evanescent wave excitation to investigate the radiations from the high-order plasmon modes excited in GNSs. The use of evanescent wave excitation makes it possible to easily control the orientation of the electric field used to excite GNSs, significantly enhancing the scattering light intensity and considerably reducing the background noise. In addition, the influence of the mirror images on the radiation behaviors of plasmon modes is also eliminated. More importantly, the radiation from the high-order plasmon modes of a GNS, such as the EQ mode, can be revealed in both the spatial and the frequency domains by exciting the GNS with *s*-polarized light and detecting the scattering light by using a *p*-polarized analyzer. The evanescent wave excitation proposed in this work is quite useful for characterizing the scattering properties of nanoparticles, especially the high-order plasmon modes. The highly directional radiations resulting from the coherent interaction of the plasmon modes may be exploited to design nanoantennas.
